# The Hospital Movement

**Published:** 1890-02

**Authors:** 


### THE HOSPITAL MOVEMENT.

The effort which has recently been inaugurated for the establishment of a city hospital in Atlanta is one which should meet with the hearty commendation and co-operation of every intelligent physician. No one can appreciate so fully as does the medical man the urgent need for such an institution. Scarcely a day passes that is not marked by the occurrence of cases which forcibly emphasize the necessity for a place where the maimed, the wounded and the sick, who have no homes where they can be properly cared for, may be placed in charge of skilful physicians and competent nurses, with all the appliances essential to successful treatment. It is no exaggeration to say that hundreds of lives are sacrificed annually for lack of such an institution.

The day is past when a hospital was a place where patients were sent as a last resort. Modern antiseptic methods have so revolutionized the results of surgical practice that erysipelas, hospital gangrene and other diseases of that class have become almost as completely extinct as the mastodon and the pterodactyl. A hospital is no longer a place to which paupers and incurables are sent to die. On the contrary it has become a well-recognized fact that a patient who has the means to command the best medical service can find no place in which a surgical operation can be so safely and successfully performed as in a well-appointed hospital. Such institutions are therefore no longer eleemosynary in character, but are to a very great extent self-supporting, since a large number of their inmates are pay patients, who add to the revenue instead of helping to exhaust it.

The city of Atlanta, in establishing a hospital, will undertake an enterprise which will draw upon its resources to a much smaller extent than is generally supposed by the laity. The first outlay for the erection of suitable buildings and their proper equipment will of course be quite large. But when once thoroughly under way it will be found that as the years go by it will become more and more self-supporting as its benefits and advantages come to be appreciated by the community. The many railroads centering in Atlanta, the many large manufacturing establishments and the many churches and benevolent societies will find it to their interest to maintain beds as the most economical way in which to care for those who are dependent upon them for medical and surgical treatment. Atlanta is the medical center for a large territory including Georgia and Florida and portions of other adjoining States. Even with our present limited facilities for the accommodation of sick strangers, the numbers who flock to this city for medical treatment would surprise any one not conversant with the facts.

After the first expenses have been met it is doubtful if the cost of maintaining a hospital would be as great to the city as the cost of supporting the sick poor under the present wretched system. But even if the expense should prove to be greater, common humanity demands that the pecuniary sacrifice should be made for the sake of the saving of human life and the amelioration of suffering which would thereby be secured.

It is a singular fact that the hospital movement has originated entirely outside of the medical profession. The impression has gone abroad that the profession is indifferent in regard to the matter. Such an impression is of course entirely erroneous and has originated in the failure of the physicians of Atlanta to agitate the subject. The united voice of the profession would have great weight with those in whose hands rests the power to decide the matter and it is important that this influence should be brought to bear in furtherance of the project. It is desirable that some concerted action should be taken, that we should "strike while the iron is hot" and not let the present effort taper out into empty nothingness as has been the case on previous occasions.

